# Prognostic Value of Neoantigen Load in Immune Checkpoint Inhibitor Therapy for Cancer

**DOI:** 10.3389/fimmu.2021.689076

**Published:** 2021-12-21

**Authors:** Xue-lin Zou, Xiao-bo Li, Hua Ke, Guang-yan Zhang, Qing Tang, Jiao Yuan, Chen-jiao Zhou, Ji-liang Zhang, Rui Zhang, Wei-yong Chen

**Affiliations:** ^1^ Department of Respiratory Medicine, Chengdu Seventh People’s Hospital, Chengdu, China; ^2^ Department of Oncology, Chengdu Seventh People’s Hospital, Chengdu, China; ^3^ Department of Thoracic Surgery, Chengdu Seventh People’s Hospital, Chengdu, China

**Keywords:** cancer, neoantigen load, immune checkpoint inhibitor, biomarker, prognostic value

## Abstract

Immune checkpoint inhibitors (ICIs) have made great progress in the field of tumors and have become a promising direction of tumor treatment. With advancements in genomics and bioinformatics technology, it is possible to individually analyze the neoantigens produced by somatic mutations of each patient. Neoantigen load (NAL), a promising biomarker for predicting the efficacy of ICIs, has been extensively studied. This article reviews the research progress on NAL as a biomarker for predicting the anti-tumor effects of ICI. First, we provide a definition of NAL, and summarize the detection methods, and their relationship with tumor mutation burden. In addition, we describe the common genomic sources of NAL. Finally, we review the predictive value of NAL as a tumor prediction marker based on various clinical studies. This review focuses on the predictive ability of NAL’s ICI efficacy against tumors. In melanoma, lung cancer, and gynecological tumors, NAL can be considered a predictor of treatment efficacy. In contrast, the use of NAL for urinary system and liver tumors requires further research. When NAL alone is insufficient to predict efficacy, its combination with other indicators can improve prediction efficiency. Evaluating the response of predictive biomarkers before the treatment initiation is essential for guiding the clinical treatment of cancer. The predictive power of NAL has great potential; however, it needs to be based on more accurate sequencing platforms and technologies.

## Introduction

Tumors acquire mutations as they develop and progress. These mutations can encode amino acid sequences to translate different proteins, called tumor-specific antigens or neoantigens, which are immunogenic and can be recognized and eliminated by immune cells (1, 2). Compared with traditional chemotherapy and targeted therapy, immune checkpoint inhibitors (ICIs) therapy have an enduring effect and efficacy in relieving the inhibitory effect of tumor cells on immune cells, thus enhancing the immune response to cancer cells. Neoantigens elicit T-cell immunoreactivity and sensitivity to ICIs (3).

Studies have shown that new epitopes avoid the effect of central T-cell tolerance, and endogenous T cells recognize new epitopes and eliminate them, making neoantigens a promising target for cancer immunotherapy (4–6). Currently, new sequencing technologies, specialized calculation methods, and the combination of human leukocyte antigen (HLA) are used to predict neoantigen load (NAL). A certain correlation between NAL and disease prognosis is an intrinsic property of neoplasms (7). A preclinical study using a UVB-induced mouse melanoma model reported that high NAL levels can predict the response probability of ICIs (8). A series of follow-up clinical studies have shown that higher NAL is associated with enhanced efficacy of ICIs in melanoma, non-small cell lung carcinoma (NSCLC), and colorectal cancer (6, 9–11).

These studies on NAL provide new directions for individualized immunotherapy. Herein, we review patients who were responsive to ICIs and had tumors with NAL expression. This review provides new insights on prognostic and predictive biomarkers of ICI sensitive cancers.

## Definition of NAL

Tumor-specific expression, such as that of somatic mutations, alternative splicing, fusion genes, non-coding RNA, and circular RNA, may produce tumor-specific antigen polypeptides (12–15). The formation of new antigens requires several steps. First, the polypeptide enters the endoplasmic reticulum (ER) through a transporter associated with the antigen processing (TAP) complex. In the ER, these peptides bind to major histocompatibility complex (MHC) class I molecules with different affinities, and the peptide-MHC class I complex is transported to the plasma membrane through the Golgi complex and recognized by CD8+ cytotoxic T cells. Although T cells can recognize antigens shared by normal and tumor cells, T-cell receptors (TCRs) usually have a higher affinity for neoantigens (16, 17). Some mutant proteins can be recognized by TCRs as neoantigens, resulting in the initiation of an immune response. The reactivity of TCRs expressed by tumor-infiltrating lymphocytes (TILs) determines their ability to interact with tumor antigens on antigen-presenting cells (APCs). Therefore, the TCR library is related to the response and survival of cancer patients to immune checkpoint blockade therapy (18, 19).

## Relationship Between NAL and Tumor Mutation Burden

The tumor mutation burden (TMB) generally refers to the number of non-synonymous mutations per megabase (Mb) of somatic cells in a specific genomic region TMB can be used to estimate the ability and tumors to produce new antigens, and has been proven to predict the efficacy of immunotherapy for a variety of tumors (1, 20). In the past, whole-exome sequencing (WES) was the first choice for TMB detection, accounting for 1% of the entire genome, including most known pathogenic mutations (21). However, its application in clinical practice is limited because of its high cost, large sample demand, and complex data analysis (20, 22–24). With the identification of a large number of tumor-related genes, the use of targeted sequencing panels for tumor genome analysis has become another option in clinical testing.

Early screening of new antigens was mainly performed using a cDNA library, however, this is a very time-consuming and laborious process. With the development of WES (25), whole-genome sequencing (26), and second-generation sequencing of the transcriptome (27), the cost of sequencing has been dramatically reduced, making it possible to quickly and effectively perform individual sequencing and neoantigen screening for each patient, thereby laying the foundation for the clinical application of NAL. In addition, large projects, such as The Cancer Genome Atlas (TCGA) (25, 28) and the International Cancer Genome Consortium (29), have identified cancer genomes across multiple tumor types. Directly excavating tumor neoantigenic epitopes in the databases and literature can identify high-frequency mutation sites in solid tumors. In addition, single-cell sequencing methods have been increasingly adopted as a high-resolution alternative method to study gene expression, genomic aberrations, microenvironment, and epigenetic modifications in the constituent cells of various malignant and benign tumors (30, 31). Overall, neoantigens play a pivotal role in cancer immunotherapy, especially in ICI therapy. The key to achieving high effective individualized immunotherapy is the development of new bioinformatics and calculation methods to improve the sensitivity and specificity of antigen identification methods.

TMB has been used as a target for predicting the efficacy of ICI therapy. Theoretically, tumor types with a high TMB often have a high predictive NAL (32). The relationship between overall TMB/NAL and ICI response in NSCLC and melanoma has been clarified in various studies (6, 9, 11). The primary explanation is that high TMB increases the formation and presentation of immune neoantigens, thereby inducing effective anti-tumor immune responses (33). Recent studies have confirmed that the higher the TMB, the higher the tumor NAL, and the more likely to a patient benefits from ICI therapy (34). It is speculated that tumors with higher mutation burden have more tumor-specific neoantigens, which stimulate the increase in the number of TILs caused by the overexpression offset of immune checkpoint modulators, such as the programmed death receptor 1 (PD-1) or programmed cell death ligand 1 (PD-L1) (35–38). ICIs can promote T cells to recognize tumors by antagonizing T-cell activation inhibitory molecules, thereby restoring the anti-tumor immune response (39).

However, TMB is not equivalent to NAL. Rizvi et al. showed that the absolute burden of candidate neoantigens, but not the frequency per non-synonymous mutation, correlated with response, suggesting the importance of neoantigens in dictating response (6). Another study analyzed the different patterns of TMB and NAL numbers in NSCLC and found that half of the oncogenic mutations did not produce neoantigens, suggesting that TMB number is not a good surrogate marker of the immunogenic neoantigen (40). TMB may thus be an indirect measure of tumor immunogenicity because somatic mutations must lead to amino acid changes in expressed proteins; thus, peptides must be presented by HLA and subsequently cause cell proliferation and kill tumors. Studies aiming to evaluate and improve the prediction of NAL when high TMB cannot effectively predict benefit from ICI therapy should be conducted in the future.

## NAL and Genomic Alterations

Currently, a large number of clinical trials have explored whether gene mutations can be used to estimate NAL to predict the response of various cancers to ICIs. The relationship between common oncogenes and NAL has been explored. Driver mutant genes may interfere with genome stability and affect immune status by generating new antigens (41). One study reported that the number of predicted neoantigens was significantly higher in BRCA1/2 mutant tumors and that tumors with higher NAL were associated with improved overall survival (OS) and higher expression of immune genes associated with tumor cytotoxicity (42). Another study found that patients with mutant TP53 (TP53-MT) showed stronger tumor antigenicity and tumor antigen presentation than patients with wild-type TP53 (TP53-WT) and were more likely to benefit from ICI therapy (43). Tran et al. found KRAS G12D mutations in lung metastasis resection tissues of patients with rectal cancer and detected polyclonal CD8+ T cells that specifically recognize KRAS G12D mutations in TILs (44).

Some rare gene mutations can also cause an increase in NAL; however, findings on the prognosis remain inconclusive. For example, Lei Zhang et al. reported that compared with patients with wild-type tumors, patients with MUC16 mutant tumors have a significant increase in NAL, which is related to improved the OS of patients with MUC16 mutation containing NSCLC and melanoma (45). Similarly, Wu et al. conducted a comprehensive analysis of patients with TET1, a DNA demethylase that regulates DNA methylation (46). They indicated that TET1 mutation was closely associated with higher NAL, presenting a higher objective response rate, better durable clinical benefit, longer progression-free survival (PFS), and improved OS in patients receiving ICIs (47). A series of studies have shown the relationship between genetic mutations and NAL, such as TP53-MT (43), Eph receptor A5 mutations (48), ZFHX3-MT (49), and AT-rich interaction domain 1A (50), which are closely related to longer OS or PFS in patients treated with ICIs.

The DNA damage response system is essential for the preservation of genomic integrity (51), and thus, mutations in this system may lead to the appearance of new alleles that are absent in normal DNA, resulting in an increase in NAL. Wang et al. demonstrated that variations in the DNA damage response pathway of homologous recombination repair (HRR), mismatch repair, and base excision repair are associated with increased NAL and increased levels of immune gene expression characteristics (52). Similarly, a study reported that a deficiency in DNA double-strand break repair, particularly HRR, is related to increased NAL on the tumor cell surface, which subsequently activates the adaptive immune response (53).

In addition, other markers can represent an increase in NAL and can thus be used as predictors of ICI efficacy. For example, the centrosome protein 78 (CEP78) is required to regulate the cell cycle (54). Huang et al. analyzed the RNA sequencing data of a muscle-invasive bladder cancer cohort and found that high CEP78 expression was correlated with high NAL; but was not associated with OS (55). A similar study has shown that the high expression of the aryl hydrocarbon receptor nuclear translocator-like protein 1 is associated with increased NAL and can be a clinically relevant biomarker for immunotherapy (56). A low m6A score was also linked to increased NAL and enhanced response to immunotherapy (57).

As more mutated genes are revealed to be clearly associated with an increase in NAL, cancer patients can be better screened for ICI therapy.

## Prognostic Value of NAL in Tumors

**Figure 1:**
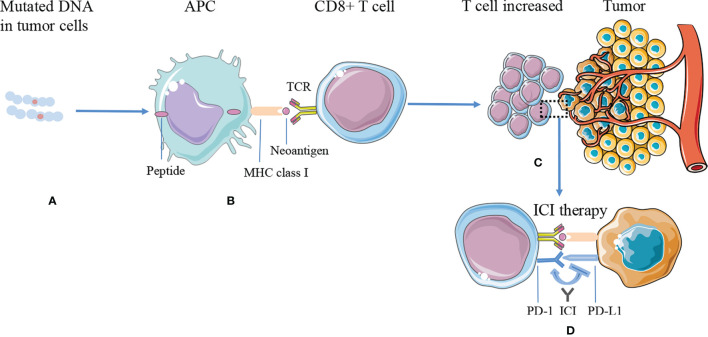
The mechanism of tumor antigen processing, presentation on MHC class I, and improving efficacy of ICI therapy. **(A)** DNA mutations occurred and synthesized proteins in the tumor cells. **(B)** The proteins are processed into smaller peptides, displayed by major histocompatibility complex (MHC) class I molecules *via* APC cells, and recognized by CD8+ T effector cells as neoantigens. **(C)** Tumors expressing higher numbers of neoantigens are more likely to induce a significantly greater number of T cells, while tumor cells inhibit T-cell function through immune checkpoints, such as PD-L1. **(D)** ICI therapy blocks immune checkpoint suppression, reactivates T-cell function, and kills tumor cells. APC, antigen-presenting cell; ICI, immune checkpoint inhibitor; MHC, major histocompatibility complex; PD-L1, programmed cell death ligand 1; PD-1, programmed death receptor 1; TCR, T-cell receptors.

The commonly used targets of immunotherapy are mainly cytotoxic T-lymphocyte-associated protein 4 (CTLA-4) or PD-1/PD-L1, which can effectively treat a variety of malignant tumors (58). Presently, it is generally believed that neoantigens and neoantigen-specific T cells are closely related to tumor regression after ICI therapy. Tumors with more mutations may produce more new epitopes, which can be recognized by tumor-infiltrating T cells. Checkpoint blocking antibodies activate these T cells in the body and induce tumor regression (59) (**Figure 1**).

These mechanisms promote the success of ICI therapy and NAL through several aspects. Tumors containing DNA repair gene mutations, such as *HR* gene, *MMR* gene, or POLE mutations, have a significantly higher mutation load and a significantly greater number of T-cell types and other anti-tumor activities than DNA repair wild-types tumors. The required immune cell infiltration increases the efficacy of ICI therapy (60). Zhu et al. analyzed the mechanism underlying the improved prognosis of NAL-high (NAL-H) and NAL-medium (NAL-M) groups. They found that the high- and medium-expression groups of NAL have significantly overexpressed genes, which are related to IFN-g/TNF-α, and are important predictors of immune activation (61). In addition, these two groups of patients have a higher degree of adaptive immune infiltration, whereas the low-expression group is enriched with innate immune infiltration. A series of studies have shown that the level of NAL can help screen this part of the patients (6, 42, 60–74) (**Table 1**).

**Table 1:** Studies describing the impact of neoantigen load evaluation in the clinical research.

Type of cancer	No of investigated patients	Test for NAL	Group	Drug/treatment	Result (whether NAL is associated with clinical benefit)	Reference
Melanoma	110	WES	High/low	Ipilimumab	YES	(62)
Melanoma	38	WES	High/low	Pembrolizumab and nivolumab	NO	(63)
Non–small cell lung cancer	34	WES	High/low	Pembrolizumab	YES	(6)
Non–small cell lung cancer	NR	RNA-sequencing	High/low	NR	YES	(60)
Non–small cell lung cancer	139	NR	High/low	PD-1 and CTLA-4 blockade	YES	(64)
Breast cancer	835	WES and RNA sequencing	high/medium/low	NR	YES	(65)
Gynecologic and breast cancers	812	RNA-sequencing	high/medium/low	Immunotherapy	YES	(61)
Endometrial cancers	150	WES	High/low	Immunotherapy	YES	(66)
Ovarian carcinoma	80	WES and RNA sequencing	High/low	Carboplatin plus paclitaxel	YES	(67)
Ovarian cancer	253	WES	High/low	PD-1/PD-L1 inhibitors	YES	(42)
Bladder tumors	37	RNA sequencing	High/low	Durvalumab	YES	(68)
Muscle-invasive Bladder Cancer	38	WES	High/low	NR	NO	(69)
Clear cell renal cell carcinoma	97	WES and RNA sequencing	High/low	Surgery alone or surgery plus cytokines tyrosine kinase inhibitors and mTOR inhibitors	NO	(70)
Clear cell renal cell carcinoma	592	WES and RNA sequencing	High/low	PD-1 blockade	NO	(71)
Multiple myeloma	184	WES and RNA sequencing	High/low	Chemotherapy, or immunotherapy	YES	(72)
Osteosarcoma	321	WES	High/low	Pembrolizumab	YES	(73)
Hepatocellular carcinoma	22	WES and RNA sequencing	High/low	Surgery alone or surgery plus chemoradiotherapy	NO	(74)

CTLA-4, cytotoxic T-lymphocyte-associated protein 4; NAL, neoantigen load; NR, not reported; PD-1, programmed death receptor 1; PD-L1, programmed cell death ligand 1; WES, whole-exome sequencing.

### NAL in Melanoma

The emergence of ICIs has completely changed the clinical management of metastatic melanoma, which has a higher mutation burden than other solid tumors (75). A clinical trial that included 110 patients with metastatic melanoma who were treated with CTLA-4 inhibitors revealed the correlation between NAL and clinical outcome, indicating that NAL could be used as a potential biomarker patients selection (62). Another study that focused on the detection of somatic mutations and the transcriptome of metastatic melanoma to identify the factors that may affect anti-PD-1 therapy found no statistical difference in NAL between reactive and non-reactive tumors; thus, the NAL of melanoma tumors before treatment was insufficient to predict the response to anti-PD-1 therapy (63).

### NAL in Lung Cancer

Neoantigens are associated with the response to anti-PD-1 therapy in patients with NSCLC (6). A previous study analyzed mutations in DNA repair genes using TCGA samples and found that NAL correlated with the expression of PD-1, PD-L1, and IFN-γ and tended to increase the OS of patients with lung adenocarcinoma. NAL is linked to DNA repair mutations, increased number of TILs, and favorable survival outcomes (60). Furthermore, advanced NSCLC treated with PD-1 and CTLA-4 blockade-based therapies was profiled for intra-tumor heterogeneity (ITH) and neoantigen burden. High NAL was associated with significantly longer OS in patients with lung adenocarcinoma. Notably, patients with homogeneous tumors (neoantigen ITH ≤1%) have a prolonged OS compared with those with heterogeneous tumors (64). Rizvi et al. analyzed NSCLC samples collected from patients treated with pembrolizumab and reported that higher NAL in tumors was associated with improved objective response, durable clinical benefit, and PFS (6).

### NAL in Gynecologic Cancer

Immunotherapy is also widely used in the field of gynecological tumors, and identifying the ideal predictive markers for therapeutic efficacy has always been the goal of researchers. A recent study developed a new informatics workflow, which was applied to detect class I and class II HLA-bound neoantigens, and reported the association between NAL and OS in breast cancer (65). A clinical study including 812 gynecologic and breast cancer patients used a NAL cutoff of 60% and 80% and divided the patients into three groups. It was found that the NAL-high and NAL-middle groups had a higher number of T cells, B cells, and cytotoxic lymphocytes, whereas the NAL-low group was rich in eosinophils, NK cells, mast cells, and interdigital cells, which represent adaptive immunity and innate immunity, respectively. Furthermore, the NAL-high group was associated with better OS, higher immune infiltration, and lower intratumoral heterogeneity (61).

A previous study showed that hypermutated POLE-mutated endometrial cancer has a higher predictive NAL and is related to its prognosis (76). Shukla et al. investigated whether low-mutation endometrial cancer has similar prognostic factors and analyzed the data of 90 copy number-low/endometrioid and 60 copy number-high/serous-like endometrial tumors using the TCGA dataset. They found that the predicted NAL was related to specific genomic changes, such as CTNNB1 mutation, MYC amplification, and PIK3CA mutation. In copy number-low/endometrioid tumors, high NAL was associated with prolonged PFS, and low NAL in serous-like endometrial tumors was associated with poor PFS (66).

Deficiencies in the homologous recombination (HR) pathway are common in high-grade serous ovarian cancer (77, 78). The best candidates for ICIs in HR-proficient ovarian cancer patients who cannot benefit from poly(ADP-ribose) polymerase inhibition have been investigated. In particilar, the exome and RNA sequencing data of 80 patients with high-grade serous ovarian cancer were analyzed. They found that the OS and PFS of the high-NAL and low-NAL groups were not statistically different. However, the inclusion of HLA class I expression status in the survival analysis showed that the subgroup of patients with high NAL and high HLA class I expression had the best PFS in HR-proficient high-grade serous ovarian cancer patients (67). Similarly, in ovarian carcinoma patients, early findings suggested that NAL is significantly associated with OS but not with PFS (42).

### NAL in Urothelial Cancer

Urothelial cancer has a high burden of somatic mutations, second only to lung cancer and melanoma (79). A study described a systematic method to effectively identify and verify immunogenic neoantigens. This method was verified in some patients with bladder tumors who received durvalumab treatment. In this cohort, the most predicted neoantigen in all patients was immunogenic *in vitro*. Finally, the patients were stratified by TMB or NAL using the three-point method to evaluate OS. The results showed that patients with higher NAL showed better OS. Although the number of included cases was small, the study demonstrated the predictive value of ICI therapy on bladder cancer; thus, future studies should examine lager cohorts (68). However, in a study of 38 muscle-invasive bladder cancer tissues from patients who underwent definitive surgery. Choudhoury et al. found that the relationship between filtered NAL and recurrence-free survival (RFS) was not statistically significant (69). Two other studies in clear-cell renal cell carcinoma have similar reported that NAL is not associated with response to ICI therapy (70, 71).

### NAL in Other Cancer

Mutations and NAL are associated with prolonged survival in patients with newly diagnosed multiple myeloma (80). In a recent study, researchers used next-generation sequencing data to describe the distribution of neoantigens in multiple myeloma and found that in patients with multiple myeloma recurrence when compared with newly diagnosed multiple myeloma patients. In this study, the neoantigen T-cell response of three patients with multiple myeloma recurrence, the NAL increased was verified and correlated with improved clinical response (72). Osteosarcoma often presents with lung metastases, and there is a lack of effective treatment strategies for it (81). Researchers have sequenced the multi-region whole exome and whole genome of 86 tumor regions of lung metastatic osteosarcoma. Metastatic tumors showed better immunogenicity, higher NAL, higher PD-L1 expression, and more TILs than primary tumors. One patient relapsed after the first primary tumor operation and subsequent lung metastasis resection. After multiple chemotherapy regimens, the patient received six cycles of pembrolizumab treatment. Lung metastases showed a partial response, and some lung metastases had disappeared, thus demonstrating that NAL may also be a potential biomarker for lung metastatic osteosarcoma (73). Yang et al. investigated neoantigens in hepatocellular carcinoma and concluded that OS was not associated with NAL (74).

## Limitations of NAL in Clinical Settings

In general, ICI therapy is not effective for all patients, and the relationship between NAL and clinical outcomes is not consistent among cancer types. However, the exact mechanisms responsible for such differences remain unclear. Here, we will disscuss some of these reasons. First, the rapid increase in the heterogeneity of tumor cells may lead to the failure of immune monitoring, thereby resulting in non-response to immunotherapy (64, 82–84). Tumors with low neoantigen ITH are associated with longer PFS (64). One possible explanation for this is that tumors with high ITH may have more neoantigens, which are subsequently presented by DCs to T cells in the form of MHC class I peptide complexes. Eventually, the ITH level in these tumors changes from high to low (85). Second, most previous studies did not comprehensively analyze the mutation types, which would result in missing data and inaccurate results. For example, they mostly included analysis of somatic non-synonymous single nucleotide mutations and small frameshift insertions and deletions but did not consider large genome rearrangements or gene fusions. The contribution of fusion genes exceeds one-third of the total NAL, and there is no correlation between gene fusion NAL and OS. Thus, this will have a significant impact on the results if there is no sufficient analysis of mutation types (65). Third, most of the analyzed data are derived from the TCGA database, wherein tumor samples are screened and excluded by pathologists. This results in a loss of information, which will inevitably affect the follow-up results (86). In addition, some researchers have proposed that the ability of neoantigens to activate T-cell recognition and the quality of T-cell responses are more important in determining the immune response during tumor evolution than the number of neoantigens. Therefore, the quality rather than the quantity of neoantigens may that affect the efficacy of ICI therapy (84). Some studies with insufficient sample sizes may not have reliable conclusions.

In addition, changes in the tumor genome landscape during ICI therapy may lead to the possible evolution of NAL and affect the efficacy of ICI therapy. Some patients who initially responded to PD-1 blockade therapy developed resistance (87). Alternate upregulation of immune checkpoints (88), loss of HLA haplotypes (89), and somatic mutations in *HLA* or *JAK1/JAK2* genes (90, 91) have been considered mechanisms by which some patients evade immune recognition. Using comprehensive genomic analysis, it was determined that the emergence of acquired drug resistance during immune checkpoint blockade therapy is related to the mutation and loss of putative tumor-specific neoantigens, including the elimination of tumor subclones or truncated changes in chromosomes (92). Several recent studies have shown that the possible mechanism for the change in the neoantigen mutation landscape during ICI treatment is the induction of tumor resistance by losing antigen or components of the antigen presentation pathway, such as b-2 microglobulin (13, 44, 91, 93, 94). Simultaneous targeting of multiple antigens or MHC class II-restricted antigens can overcome this resistance. In addition, the combination of checkpoint blocking therapy and T-cell therapy can prevent T-cell failure and improve clinical efficacy (95).

To more accurately predict the therapeutic effect, NAL combined with other indicators is a feasible method of testing. A recent study reported no difference between the high-NAL and the low-NAL groups; however, according to the subgroup analysis results, the high-NAL and the high- HLA-I expression groups were associated with better PFS than the other groups (67). Similarly, in a clear-cell renal cell carcinoma cohort analyzed by Matsushita et al., the high level of NAL combined with the number of HLA-restricted neoepitopes correlated with better clinical outcomes (70). Another study demonstrated that NAL was not correlated with RFS, however, patients with more neoantigens and low T-cell receptor β diversity had a prolonged RFS compared with those with fewer neoantigens and high TCR diversity (69). Therefore, when NAL alone cannot be used to predict efficacy, the combined test will facilitate the application of NAL and improve prediction efficiency.

## Conclusions

ICIs provide cancer patients with more options, in addition to targeted therapy drugs. However, the effectiveness of this treatment is not satisfactory and many patients do not benefit from it. The exploration of effective curative predictors is currently ongoing, and NAL has a promising as a new generation of ICI biomarkers. With rapid advancements in sequencing technologies, NAL can become more reliable markers. NAL alone or in combination with other indicators can provide accurate clinical guidance for patients receiving immunotherapy.

## Author Contributions

X-bL and W-yC: conceptualization. X-lZ, G-yZ, and HK: original draft writing. JY, QT, C-jZ, J-lZ, and RZ: manuscript review and editing. All authors contributed to the article and approved the submitted version.

## Funding

This work was supported by the 2020 Chengdu Medical College & Chengdu Seventh People's Hospital Joint Scientific Research Fund (grant number 2020LHJYPJ-05).

## Conflict of Interest

The authors declare that the research was conducted in the absence of any commercial or financial relationships that could be construed as a potential conflict of interest.

## Publisher’s Note

All claims expressed in this article are solely those of the authors and do not necessarily represent those of their affiliated organizations, or those of the publisher, the editors and the reviewers. Any product that may be evaluated in this article, or claim that may be made by its manufacturer, is not guaranteed or endorsed by the publisher.

## References

[1] Schumacher TN Schreiber RD. Neoantigens in Cancer Immunotherapy. Science (2015) 6230:69–74. doi: 10.1126/science.aaa4971 25838375

[2] Castle JC Uduman M Pabla S Stein RB Buell JS. Mutation-Derived Neoantigens for Cancer Immunotherapy. Front Immunol (2019) 10:1856. doi: 10.3389/fimmu.2019.01856 31440245 PMC6693295

[3] Brown SD Warren RL Gibb EA Martin SD Spinelli JJ Nelson BH. Neo-Antigens Predicted by Tumor Genome Meta-Analysis Correlate With Increased Patient Survival. Genome Res (2014) 5:743–50. doi: 10.1101/gr.165985.113 PMC4009604 24782321

[4] Lennerz V Fatho M Gentilini C Frye RA Lifke A Ferel D. The Response of Autologous T Cells to a Human Melanoma is Dominated by Mutated Neoantigens. Proc Natl Acad Sci USA (2005) 44:16013–8. doi: 10.1073/pnas.0500090102 PMC1266037 16247014

[5] Ostroumov D Fekete-Drimusz N Saborowski M K¨¹hnel F Woller N. CD4 and CD8 T Lymphocyte Interplay in Controlling Tumor Growth. Cell Mol Life Sci (2018) 4:689–713. doi: 10.1007/s00018-017-2686-7 PMC5769828 29032503

[6] Rizvi NA Hellmann MD Snyder A Kvistborg P Makarov V Havel JJ. Cancer Immunology. Mutational Landscape Determines Sensitivity to PD-1 Blockade in non-Small Cell Lung Cancer. Science (2015) 6230:124–8. doi: 10.1126/science.aaa1348 PMC4993154 25765070

[7] Samstein RM Lee CH Shoushtari AN Hellmann MD Shen R Janjigian YY. Tumor Mutational Load Predicts Survival After Immunotherapy Across Multiple Cancer Types. Nat Genet (2019) 2:202–06. doi: 10.1038/s41588-018-0312-8 PMC6365097 30643254

[8] Wang J Perry CJ Meeth K Thakral D Damsky W Micevic G. UV-Induced Somatic Mutations Elicit a Functional T Cell Response in the YUMMER1.7 Mouse Melanoma Mode L. Pigment Cell Melanoma Res (2017) 4:428–35. doi: 10.1111/pcmr.12591 PMC5820096 28379630

[9] Snyder A Makarov V Merghoub T Yuan J Zaretsky JM Desrichard A. Genetic Basis for Clinical Response to CTLA-4 Blockade in Melanoma. N Engl J Med (2014) 23:2189–99. doi: 10.1056/NEJMoa1406498 PMC4315319 25409260

[10] Le DT Uram JN Wang H Bartlett BR Kemberling H Eyring AD. PD-1 Blockade in Tumors With Mismatch-Repair Deficiency. N Engl J Med (2015) 26:2509–20. doi: 10.1056/NEJMoa1500596 PMC4481136 26028255

[11] Hugo W Zaretsky JM Sun L Song C Moreno BH Hu-Lieskovan S. Genomic and Transcriptomic Features of Response to Anti-PD-1 Therapy in Metastatic Melanoma. Cell (2016) 1:35–44. doi: 10.1016/j.cell.2016.02.065 PMC4808437 26997480

[12] Zhang M Fritsche J Roszik J Williams LJ Peng X Chiu Y. RNA Editing Derived Epitopes Function as Cancer Antigens to Elicit Immune Responses. Nat Commun (2018) 1:3919. doi: 10.1038/s41467-018-06405-9 PMC6156571 30254248

[13] Sahin U Derhovanessian E Miller M Kloke B-P Simon P Löwer M. Personalized RNA Mutanome Vaccines Mobilize Poly-Specific Therapeutic Immunity Against Cancer. Nature (2017) 7662:222–26. doi: 10.1038/nature23003 28678784

[14] Smith CC Selitsky SR Chai S Armistead PM Vincent BG Serody JS. Alternative Tumour-Specific Antigens. Nat Rev Cancer. (2019) 8:465–78. doi: 10.1038/s41568-019-0162-4 PMC6874891 31278396

[15] Yang W Lee KW Srivastava RM Kuo F Krishna C Chowell D. Immunogenic Neoantigens Derived From Gene Fusions Stimulate T Cell Responses. Nat Med (2019) 5:767–75. doi: 10.1038/s41591-019-0434-2 PMC6558662 31011208

[16] Melero I Gaudernack G Gerritsen W Huber C Parmiani G Scholl S. Therapeutic Vaccines for Cancer: An Overview of Clinical Trials. Nat Rev Clin Oncol (2014) 9:509–24. doi: 10.1038/nrclinonc.2014.111 25001465

[17] Aleksic M Liddy N Molloy PE Pumphrey N Vuidepot A Chang KM. Different Affinity Windows for Virus and Cancer-Specific T-Cell Receptors: Implications for Therapeutic Strategies. Eur J Immunol (2012) 12:3174–9. doi: 10.1002/eji.201242606 PMC3776049 22949370

[18] Tumeh PC Harview CL Yearley JH Shintaku IP Taylor EJ Robert L. PD-1 Blockade Induces Responses by Inhibiting Adaptive Immune Resistance. Nature (2014) 7528:568–71. doi: 10.1038/nature13954 PMC4246418 25428505

[19] Cha E Klinger M Hou Y Cummings C Ribas A Faham M. Improved Survival With T Cell Clonotype Stability After Anti-CTLA-4 Treatment in Cancer Patients. Sci Transl Med (2014) 238:238ra70. doi: 10.1126/scitranslmed.3008211 PMC4558099 24871131

[20] Chalmers ZR Connelly CF Fabrizio D Gay L Ali SM Ennis R. Analysis of 100,000 Human Cancer Genomes Reveals the Landscape of Tumor Mutational Burden. Genome Med (2017) 1:34. doi: 10.1186/s13073-017-0424-2 PMC5395719 28420421

[21] Ng SB Turner EH Robertson PD Flygare SD Bigham AW Lee C. Targeted Capture and Massively Parallel Sequencing of 12 Human Exomes. Nature (2009) 7261:272–6. doi: 10.1038/nature08250 PMC2844771 19684571

[22] Viale G Trapani D Curigliano G. Mismatch Repair Deficiency as a Predictive Biomarker for Immunotherapy Efficacy. BioMed Res Int (2017) 2017:4719194. doi: 10.1155/2017/4719194 28770222 PMC5523547

[23] Frampton GM Fichtenholtz A Otto GA Wang K Downing SR He J. Development and Validation of a Clinical Cancer Genomic Profiling Test Based on Massively Parallel DNA Sequencing. Nat Biotechnol (2013) 11:1023–31. doi: 10.1038/nbt.2696 PMC5710001 24142049

[24] Campesato LF Barroso-Sousa R Jimenez L Correa BR Sabbaga J Hoff PM. Comprehensive Cancer-Gene Panels can be Used to Estimate Mutational Load and Predict Clinical Benefit to PD-1 Blockade in Clinical Practice. Oncotarget (2015) 33:34221–7. doi: 10.18632/oncotarget.5950 PMC4741447 26439694

[25] Garraway LA Lander ES. Lessons From the Cancer Genome. Cell (2013) 1:17–37. doi: 10.1016/j.cell.2013.03.002 23540688

[26] Meyerson M Gabriel S Getz G. Advances in Understanding Cancer Genomes Through Second-Generation Sequencing. Nat Rev Genet (2010) 10:685–96. doi: 10.1038/nrg2841 20847746

[27] Morrissy AS Morin RD Delaney A Zeng T McDonald H Jones S. Next-Generation Tag Sequencing for Cancer Gene Expression Profiling. Genome Res (2009) 10:1825–35. doi: 10.1101/gr.094482.109 PMC2765282 19541910

[28] Cancer Genome Atlas Research N. Comprehensive Genomic Characterization Defines Human Glioblastoma Genes and Core Pathways. Nature (2008) 7216:1061–8. doi: 10.1038/nature07385 PMC2671642 18772890

[29] International Cancer Genome C Hudson TJ Anderson W Artez A Barker AD Bell C. International Network of Cancer Genome Projects. Nature (2010) 7291:993–8. doi: 10.1038/nature08987 PMC2902243 20393554

[30] Su F Zhang W Zhang D Zhang Y Pang C Huang Y. Spatial Intratumor Genomic Heterogeneity Within Localized Prostate Cancer Revealed by Single-Nucleus Sequencing. Eur Urol (2018) 5:551–59. doi: 10.1016/j.eururo.2018.06.005 29941308

[31] Zhang Y Narayanan SP Mannan R Raskind G Wang X Vats P. Single-Cell Analyses of Renal Cell Cancers Reveal Insights Into Tumor Microenvironment, Cell of Origin, and Therapy Response. Proc Natl Acad Sci USA (2021) 24:e2103240118. doi: 10.1073/pnas.2103240118 PMC8214680 34099557

[32] Howitt BE Shukla SA Sholl LM Ritterhouse LL Watkins JC Rodig S. Association of Polymerase E-Mutated and Microsatellite-Instable Endometrial Cancers With Neoantigen L Oad, Number of Tumor-Infiltrating Lymphocytes, and Expression of PD-1 and PD-L1. JAMA Oncol (2015) 9:1319–23. doi: 10.1001/jamaoncol.2015.2151 26181000

[33] Maleki Vareki S. High and Low Mutational Burden Tumors Versus Immunologically Hot and Cold Tumors and Response to Immu Ne Checkpoint Inhibitors. J Immunother Cancer (2018) 1:157. doi: 10.1186/s40425-018-0479-7 PMC6307306 30587233

[34] Büttner R Longshore JW López-Ríos F Merkelbach-Bruse S Normanno N Rouleau E. Implementing TMB Measurement in Clinical Practice: Considerations on Assay Requirements. ESMO Open (2019) 1:e000442. doi: 10.1136/esmoopen-2018-000442 PMC6350758 30792906

[35] Xiao Y Freeman GJ. The Microsatellite Instable Subset of Colorectal Cancer is a Particularly Good Candidate for Checkpoint Blockade Immunotherapy. Cancer Discov (2015) 1:16–8. doi: 10.1158/2159-8290.Cd-14-1397 PMC4295637 25583798

[36] Llosa NJ Cruise M Tam A Wicks EC Hechenbleikner EM Taube JM. The Vigorous Immune Microenvironment of Microsatellite Instable Colon Cancer is Balanced by Multiple Counter-Inhibitory Checkpoints. Cancer Discov (2015) 1:43–51. doi: 10.1158/2159-8290.Cd-14-0863 PMC4293246 25358689

[37] Hussein YR Weigelt B Levine DA Schoolmeester JK Dao LN Balzer BL. Clinicopathological Analysis of Endometrial Carcinomas Harboring Somatic POLE Exonuclease Domain Mutations. Mod Pathol (2015) 4:505–14. doi: 10.1038/modpathol.2014.143 25394778

[38] Rooney MS Shukla SA Wu CJ Getz G Hacohen N. Molecular and Genetic Properties of Tumors Associated With Local Immune Cytolytic Activity. Cell (2015) 1-2:48–61. doi: 10.1016/j.cell.2014.12.033 PMC4856474 25594174

[39] Chabanon RM Pedrero M Lefebvre C Marabelle A Soria JC Postel-Vinay S. Mutational Landscape and Sensitivity to Immune Checkpoint Blockers. Clin Cancer Res (2016) 17:4309–21. doi: 10.1158/1078-0432.Ccr-16-0903 27390348

[40] Gong L He R Xu Y Luo T Jin K Yuan W. Neoantigen Load as a Prognostic and Predictive Marker for Stage II/III Non-Small Cell Lung Cancer in Chinese Patients. Thorac Cancer (2021) 15:2170–81. doi: 10.1111/1759-7714.14046 PMC8327700 34128337

[41] Markowitz SD Bertagnolli MM. Molecular Origins of Cancer: Molecular Basis of Colorectal Cancer. N Engl J Med (2009) 25:2449–60. doi: 10.1056/NEJMra0804588 PMC2843693 20018966

[42] Strickland KC Howitt BE Shukla SA Rodig S Ritterhouse LL Liu JF. Association and Prognostic Significance of BRCA1/2-Mutation Status With Neoantigen Load, Number of Tumor-Infiltrating Lymphocytes and Expression of PD-1/PD-L1 in High Grade Serous Ovarian Cancer. Oncotarget (2016) 12:13587–98. doi: 10.18632/oncotarget.7277 PMC4924663 26871470

[43] Lyu Q Lin A Cao M Xu A Luo P Zhang J. Alterations in TP53 Are a Potential Biomarker of Bladder Cancer Patients Who Benefit From Immune Checkpoint Inhibition. Cancer Control (2020) 1:1073274820976665. doi: 10.1177/1073274820976665 PMC8480364 33356494

[44] Tran E Robbins PF Lu YC Prickett TD Gartner JJ Jia L. T-Cell Transfer Therapy Targeting Mutant KRAS in Cancer. N Engl J Med (2016) 23:2255–62. doi: 10.1056/NEJMoa1609279 PMC5178827 27959684

[45] Zhang L Han X Shi Y. Association of MUC16 Mutation With Response to Immune Checkpoint Inhibitors in Solid Tumors. JAMA Netw Open (2020) 8:e2013201. doi: 10.1001/jamanetworkopen.2020.13201 PMC7450349 32845327

[46] Williams K Christensen J Pedersen MT Johansen JV Cloos PA Rappsilber J. TET1 and Hydroxymethylcytosine in Transcription and DNA Methylation Fidelity. Nature (2011) 7347:343–8. doi: 10.1038/nature10066 PMC3408592 21490601

[47] Wu HX Chen YX Wang ZX Zhao Q He MM Wang YN. Alteration in TET1 as Potential Biomarker for Immune Checkpoint Blockade in Multiple Cancers. J Immunother Cancer (2019) 1:264. doi: 10.1186/s40425-019-0737-3 PMC6798429 31623662

[48] Huang W Lin A Luo P Liu Y Xu W Zhu W. EPHA5 Mutation Predicts the Durable Clinical Benefit of Immune Checkpoint Inhibitors in Patients With Lung Adenocarcinoma. Cancer Gene Ther (2020) 28:864–74. doi: 10.21203/rs.3.rs-27361/v1 32759987

[49] Zhang J Zhou N Lin A Luo P Chen X Deng H. ZFHX3 Mutation as a Protective Biomarker for Immune Checkpoint Blockade in non-Small Cell Lung Cancer. Cancer Immunol Immunother (2021) 1:137–51. doi: 10.1007/s00262-020-02668-8 PMC10992006 32653938

[50] Li Z Lin J Zhang L Li J Zhang Y Zhao C. Comprehensive Analysis of Multiple Parameters Associated With Tumor Immune Microenvironment in ARID1A Mutant Cancers. Future Oncol (2020) 29:2295–306. doi: 10.2217/fon-2020-0243 32639175

[51] Lee JK Choi YL Kwon M Park PJ. Mechanisms and Consequences of Cancer Genome Instability: Lessons From Genome Sequencing Studies. Annu Rev Pathol (2016) 283–312. doi: 10.1146/annurev-pathol-012615-044446 26907526

[52] Wang Z Zhao J Wang G Zhang F Zhang Z Zhang F. Comutations in DNA Damage Response Pathways Serve as Potential Biomarkers for Immune Checkpoint Block Ade. Cancer Res (2018) 22:6486–96. doi: 10.1158/0008-5472.CAN-18-1814 30171052

[53] Mouw KW D'Andrea AD. DNA Repair Deficiency and Immunotherapy Response. J Clin Oncol (2018) 17:1710–13. doi: 10.1200/JCO.2018.78.2425 29683789

[54] Kumar A Rajendran V Sethumadhavan R Purohit R. CEP Proteins: The Knights of Centrosome Dynasty. Protoplasma (2013) 5:965–83. doi: 10.1007/s00709-013-0488-9 23456457

[55] Huang X Yan Y Wei R Liu H Zhu X Bi D. Centrosome Protein 78 Is Overexpressed in Muscle-Invasive Bladder Cancer and Is Associated With Tumor Molecular Subtypes and Mutation Signatures. Med Sci Monit (2020) 26:e925197. doi: 10.12659/MSM.925197 33119552 PMC7607667

[56] de Assis LVM Kinker GS Moraes MN Markus RP Fernandes PA Castrucci AML. Expression of the Circadian Clock Gene BMAL1 Positively Correlates With Antitumor Immunity and Patien T Survival in Metastatic Melanoma. Front Oncol (2018) 8:185. doi: 10.3389/fonc.2018.00185 29946530 PMC6005821

[57] Zhang B Wu Q Li B Wang D Wang L Zhou YL. M6a Regulator-Mediated Methylation Modification Patterns and Tumor Microenvironment Infiltration Char Acterization in Gastric Cancer. Mol Cancer (2020) 1:53. doi: 10.1186/s12943-020-01170-0 PMC7066851 32164750

[58] Callahan MK Postow MA Wolchok JD. Targeting T Cell Co-Receptors for Cancer Therapy. Immunity (2016) 5:1069–78. doi: 10.1016/j.immuni.2016.04.023 27192570

[59] Robbins PF Morgan RA Feldman SA Yang JC Sherry RM Dudley ME. Tumor Regression in Patients With Metastatic Synovial Cell Sarcoma and Melanoma Using Genetically Engineered Lymphocytes Reactive With NY-ESO-1. J Clin Oncol (2011) 7:917–24. doi: 10.1200/jco.2010.32.2537 PMC3068063 21282551

[60] Chae YK Anker JF Bais P Namburi S Giles FJ Chuang JH. Mutations in DNA Repair Genes are Associated With Increased Neo-Antigen Load and Activated T Cell Infiltration in Lung Adenocarcinoma. Oncotarget (2018) 8:7949–60. doi: 10.18632/oncotarget.23742 PMC5814272 29487705

[61] Zhu Y Meng X Ruan X Lu X Yan F Wang F. Characterization of Neoantigen Load Subgroups in Gynecologic and Breast Cancers. Front Bioeng Biotechnol (2020) 702:702. doi: 10.3389/fbioe.2020.00702 PMC7370692 32754579

[62] Van Allen EM Miao D Schilling B Shukla SA Blank C Zimmer L. Genomic Correlates of Response to CTLA-4 Blockade in Metastatic Melanoma. Science (2015) 6257:207–11. doi: 10.1126/science.aad0095 PMC5054517 26359337

[63] Aguadé-Gorgorió G Solé R. Tumour Neoantigen Heterogeneity Thresholds Provide a Time Window for Combination Immunotherapy. J R Soc Interface (2020) 171:20200736. doi: 10.1098/rsif.2020.0736 PMC7653380 33109023

[64] McGranahan N Furness AJ Rosenthal R Ramskov S Lyngaa R Saini SK. Clonal Neoantigens Elicit T Cell Immunoreactivity and Sensitivity to Immune Checkpoint Blockade. Science (2016) 6280:1463–9. doi: 10.1126/science.aaf1490 PMC4984254 26940869

[65] Ren Y Cherukuri Y Wickland DP Sarangi V Tian S Carter JM. HLA Class-I and Class-II Restricted Neoantigen Loads Predict Overall Survival in Breast Cancer. Oncoimmunology (2020) 1:1744947. doi: 10.1080/2162402x.2020.1744947 PMC7255108 32523802

[66] Shukla SA Howitt BE Wu CJ Konstantinopoulos PA. Predicted Neoantigen Load in non-Hypermutated Endometrial Cancers: Correlation With Outcome and Tumor-Specific Genomic Alterations. Gynecol Oncol Rep (2017) 19:42–5. doi: 10.1016/j.gore.2016.12.009 PMC5219603 28070553

[67] Matsushita H Hasegawa K Oda K Yamamoto S Asada K Karasaki T. Neoantigen Load and HLA-Class I Expression Identify a Subgroup of Tumors With a T-Cell-Inflamed Phenotype and Favorable Prognosis in Homologous Recombination-Proficient High-Grade Serous Ovarian Carcinoma. J Immunother Cancer (2020) 1:e000375. doi: 10.1136/jitc-2019-000375 PMC7254153 32461346

[68] Lazdun Y Si H Creasy T Ranade K Higgs BW Streicher K. A New Pipeline to Predict and Confirm Tumor Neoantigens Predict Better Response to Immune Checkpoint Blockade. Mol Cancer Res (2021) 3:498–506. doi: 10.1158/1541-7786.Mcr-19-1118 33257508

[69] Choudhury NJ Kiyotani K Yap KL Campanile A Antic T Yew PY. Low T-Cell Receptor Diversity, High Somatic Mutation Burden, and High Neoantigen Load as Predictors of Clinical Outcome in Muscle-Invasive Bladder Cancer. Eur Urol Focus (2016) 4:445–52. doi: 10.1016/j.euf.2015.09.007 28723478

[70] Matsushita H Sato Y Karasaki T Nakagawa T Kume H Ogawa S. Neoantigen Load, Antigen Presentation Machinery, and Immune Signatures Determine Prognosis in Clear Cell Renal Cell Carcinoma. Cancer Immunol Res (2016) 5:463–71. doi: 10.1158/2326-6066.Cir-15-0225 26980598

[71] Braun DA Hou Y Bakouny Z Ficial M Sant' Angelo M Forman J. Interplay of Somatic Alterations and Immune Infiltration Modulates Response to PD-1 Blockade in Advanced Clear Cell Renal Cell Carcinoma. Nat Med (2020) 6:909–18. doi: 10.1038/s41591-020-0839-y PMC7499153 32472114

[72] Perumal D Imai N Laganà A Finnigan J Melnekoff D Leshchenko VV. Mutation-Derived Neoantigen-Specific T-Cell Responses in Multiple Myeloma. Clin Cancer Res (2020) 2:450–64. doi: 10.1158/1078-0432.Ccr-19-2309 PMC6980765 31857430

[73] Wang D Niu X Wang Z Song CL Huang Z Chen KN. Multiregion Sequencing Reveals the Genetic Heterogeneity and Evolutionary History of Osteosarcoma and Matched Pulmonary Metastases. Cancer Res (2019) 1:7–20. doi: 10.1158/0008-5472.CAN-18-1086 30389703

[74] Yang H Sun L Guan A Yin H Liu M Mao X. Unique TP53 Neoantigen and the Immune Microenvironment in Long-Term Survivors of Hepatocellular Carci Noma. Cancer Immunol Immunother (2021) 3:667–77. doi: 10.1007/s00262-020-02711-8 PMC10992148 32876735

[75] Alexandrov LB Nik-Zainal S Wedge DC Aparicio SA Behjati S Biankin AV. Signatures of Mutational Processes in Human Cancer. Nature (2013) 7463:415–21. doi: 10.1038/nature12477 PMC3776390 23945592

[76] Cancer Genome Atlas Research N Kandoth C Schultz N Cherniack AD Akbani R Liu Y. Integrated Genomic Characterization of Endometrial Carcinoma. Nature (2013) 7447:67–73. doi: 10.1038/nature12325 PMC3704730 23636398

[77] Konstantinopoulos PA Ceccaldi R Shapiro GI D'Andrea AD. Homologous Recombination Deficiency: Exploiting the Fundamental Vulnerability of Ovarian Cancer. Cancer Discov (2015) 11:1137–54. doi: 10.1158/2159-8290.CD-15-0714 PMC4631624 26463832

[78] Frey MK Pothuri B. Homologous Recombination Deficiency (HRD) Testing in Ovarian Cancer Clinical Practice: A Review of Th E Literature. Gynecol Oncol Res Pract (2017) 4:4. doi: 10.1186/s40661-017-0039-8 28250960 PMC5322589

[79] Cancer Genome Atlas Research N. Comprehensive Molecular Characterization of Urothelial Bladder Carcinoma. Nature (2014) 7492:315–22. doi: 10.1038/nature12965 PMC3962515 24476821

[80] Miller A Asmann Y Cattaneo L Braggio E Keats J Auclair D. High Somatic Mutation and Neoantigen Burden are Correlated With Decreased Progression-Free Survival in Multiple Myeloma. Blood Cancer J (2017) 9:e612. doi: 10.1038/bcj.2017.94 PMC5709757 28937974

[81] Reed DR Hayashi M Wagner L Binitie O Steppan DA Brohl AS. Treatment Pathway of Bone Sarcoma in Children, Adolescents, and Young Adults. Cancer (2017) 12:2206–18. doi: 10.1002/cncr.30589 PMC5485018 28323337

[82] Wolf Y Bartok O Patkar S Eli GB Cohen S Litchfield K. UVB-Induced Tumor Heterogeneity Diminishes Immune Response in Melanoma. Cell (2019) 1:219–35.e21. doi: 10.1016/j.cell.2019.08.032 PMC6863386 31522890

[83] Lakatos E Williams MJ Schenck RO Cross WCH Househam J Zapata L. Evolutionary Dynamics of Neoantigens in Growing Tumors. Nat Genet (2020) 10:1057–66. doi: 10.1038/s41588-020-0687-1 PMC7610467 32929288

[84] uksza M Riaz N Makarov V Balachandran VP Hellmann MD Solovyov A. A Neoantigen Fitness Model Predicts Tumour Response to Checkpoint Blockade Immunotherapy. Nature (2017) 7681:517–20. doi: 10.1038/nature24473 PMC6137806 29132144

[85] Yewdell JW Dersh D Fåhraeus R. Peptide Channeling: The Key to MHC Class I Immunosurveillance? Trends Cell Biol (2019) 12:929–39. doi: 10.1016/j.tcb.2019.09.004 31662235

[86] Berger AC Korkut A Kanchi RS Hegde AM Lenoir W Liu W. A Comprehensive Pan-Cancer Molecular Study of Gynecologic and Breast Cancers. Cancer Cell (2018) 4:690–705.e9. doi: 10.1158/1538-7445.AM2018-3303 PMC5959730 29622464

[87] Gettinger SN Horn L Gandhi L Spigel DR Antonia SJ Rizvi NA. Overall Survival and Long-Term Safety of Nivolumab (Anti-Programmed Death 1 Antibody, BMS-936558, ONO-4538) in Patients With Previously Treated Advanced Non-Small-Cell Lung Cancer. J Clin Oncol (2015) 18:2004–12. doi: 10.1200/jco.2014.58.3708 PMC4672027 25897158

[88] Koyama S Akbay EA Li YY Herter-Sprie GS Buczkowski KA Richards WG. Adaptive Resistance to Therapeutic PD-1 Blockade is Associated With Upregulation of Alternative Immune Checkpoints. Nat Commun (2016) 7:10501. doi: 10.1038/ncomms10501 26883990 PMC4757784

[89] Maeurer MJ Gollin SM Storkus WJ Swaney W Karbach J Martin D. Tumor Escape From Immune Recognition: Loss of HLA-A2 Melanoma Cell Surface Expression is Associated With a Complex Rearrangement of the Short Arm of Chromosome 6. Clin Cancer Res (1996) 4:641–52. 9816214

[90] Shukla SA Rooney MS Rajasagi M Tiao G Dixon PM Lawrence MS. Comprehensive Analysis of Cancer-Associated Somatic Mutations in Class I HLA Genes. Nat Biotechnol (2015) 11:1152–8. doi: 10.1038/nbt.3344 PMC4747795 26372948

[91] Zaretsky JM Garcia-Diaz A Shin DS Escuin-Ordinas H Hugo W Hu-Lieskovan S. Mutations Associated With Acquired Resistance to PD-1 Blockade in Melanoma. N Engl J Med (2016) 9:819–29. doi: 10.1056/NEJMoa1604958 PMC5007206 27433843

[92] Anagnostou V Smith KN Forde PM Niknafs N Bhattacharya R White J. Evolution of Neoantigen Landscape During Immune Checkpoint Blockade in Non-Small Cell Lung Cancer. Cancer Discov (2017) 3:264–76. doi: 10.1158/2159-8290.Cd-16-0828 PMC5733805 28031159

[93] Ott PA Hu Z Keskin DB Shukla SA Sun J Bozym DJ. An Immunogenic Personal Neoantigen Vaccine for Patients With Melanoma. Nature (2017) 7662:217–21. doi: 10.1038/nature22991 PMC5577644 28678778

[94] Riaz N Havel JJ Makarov V Desrichard A Urba WJ Sims JS. Tumor and Microenvironment Evolution During Immunotherapy With Nivolumab. Cell (2017) 4:934–49.e16. doi: 10.1016/j.cell.2017.09.028 PMC5685550 29033130

[95] Lu YC Zheng Z Robbins PF Tran E Prickett TD Gartner JJ. An Efficient Single-Cell RNA-Seq Approach to Identify Neoantigen-Specific T Cell Receptors. Mol Ther (2018) 2:379–89. doi: 10.1016/j.ymthe.2017.10.018 PMC5835023 29174843

